# Pamphlets and Books Received

**Published:** 1884-01-20

**Authors:** 


### PAMPHLETS & BOOKS RECEIVED.

Index to Transactions of the American Medical Association, Vols. I-XXXIII. Prepared by Wm. D. Atkinson, M.D., Secretary. Philadelphia: Wm. F. Few & Co., 1883.

Physician's Pocket Day-Book, designed by C. Henri Leonard, M.A., M.D., Professor of Medical and Surgical Diseases of Women and Clinical Gynecology in Mich. College of Medicine, etc., Detroit, Mich.

An excellent pocket memorandum. Has blanks for twenty to forty families, etc., etc—very useful. Price, $1 to $1.25.

*The Physician's Visiting List for* 1884—Thirty-third year of its publication—well known to the profession, and excellent in its get up and arrangement for the medical practitioner. Philadelphia: P, Blakiston, Son & Co. Price, $1.00 to $3.00, according to size.

